# Perfect appearance match between self-luminous and surface colors can be performed with isomeric spectra

**DOI:** 10.1038/s41598-020-75510-x

**Published:** 2020-10-27

**Authors:** Akari Kagimoto, Katsunori Okajima

**Affiliations:** 1 Graduate School of Environment and Information Sciences, Yokohama National University, Yokohama, 240-8501 Japan; 2 Faculty of Environment and Information Sciences, Yokohama National University, Yokohama, 240-8501 Japan

**Keywords:** Displays, Colour vision

## Abstract

Surface color results from a reflected light bounced off a material, such as a paper. By contrast, self-luminous color results directly from an emitting light, such as a Liquid Crystal (LC) display. These are completely different mechanisms, and thus, surface color and self-luminous color cannot be matched even though both have identical tristimulus values. In fact, previous research has reported that metameric color matching fails among diverse media. However, the reason for this failure remains unclear. In the present study, we created isomeric color-matching pairs between self-luminous and surface colors by modulating the spectral distribution of the light for surface colors. Then, we experimentally verified whether such color matching can be performed. The results show that isomeric color matching between self-luminous and surface colors can be performed for all participants. However, metameric color matching fails for most participants, indicating that differences in the spectral distributions rather than the different color-generating mechanisms themselves are the reason for the color matching failure between different devices. We experimentally demonstrated that there is no essential problem in cross-media color matching by generating isomeric pairs. Our results can be considered to be of great significance not only for color science, but also for the color industry.

## Introduction

Colors are broadly classified as surface and self-luminous colors. Surface color refers to the color that is created by a reflected light that bounces off an object, such as colors on paper, wood, and fabric. Conversely, self-luminous color refers to the color created by the devices’ own lights, such as Liquid Crystal (LC) Display, Organic Light Emitting Diode (OLED), and CRT displays. In colorimetry, a color is numerically expressed by tristimulus values that can be calculated using the color matching functions (CMFs), the spectral power of a luminaire for self-luminous color, or the spectral reflectivity of an object and illumination for the surface color.

Theoretically, the color appearances of lights that have equal colorimetric values should match regardless of their spectral distributions. Such a phenomenon is called “metamerism,” which is the foundation of color science and technology. However, the color appearances of objects with the same colorimetric values on a monitor and a paper are mostly mismatched^1–10^, even under conditions where not only the tristimulus values of the targets but also those of the surrounding environment and observation environment are identical^11–13^.

Three possible causes have been considered for this phenomenon. First, the physical effect is different. As previously noted, surface and self-luminous colors result from different generating mechanisms. For example, the color on printed paper is represented by a subtractive mixture of *Cyan, Magenta, Yellow* and *Black*, and we recognize the color by the light that bounces off the print. By contrast, the display color on a monitor is represented by an additive mixture of the three primary *Red, Green* and *Blue* phosphors that are used to coat the screen and emit light by themselves; we recognize a color by the light that enters our eyes directly. However, the quantitative reason for this is unclear. Second, the phenomenon could be caused by individual CMFs’ variability. It has been shown that the range of differences caused by observer variability related to the difference in color appearance is very large^12,13^. In addition, human eye lens density increases with age^14^. Because common CMFs are composed of average values, people with different CMFs are not expected to see objects as having the same color appearance even if the colorimetric values are the same. However, the color appearances cannot be matched even when the stimuli are created considering the individual CMFs of the observers^15^. Therefore, we should take into account considerations other than the individual differences of CMFs. Finally, there may be influences other than those of the L-, M-, and S-cones of the eye. In 2000, melanopsin-expressing intrinsically photosensitive retinal ganglion cells (ipRGCs) were detected in cells of the mammalian inner retina^16^. They have been reported to significantly influence non-image-forming vision, for example, in melatonin suppression^17,18^, circadian entrainment^19,20^, and pupillary reflex^21,22^. However, recent studies have shown that ipRGCs also project to the lateral geniculate nucleus^23–26^. This suggests that melanopsin influences cortical vision, including color perception. In addition, some reports have suggested a rod intrusion to the color perception^27–29^ in both foveal and peripheral vision^30^. According to an electrophysiological recording study, rods contribute to visual responses in the photopic range^31^. Therefore, these photoreceptive cells may also be related to the color appearance. In fact, ipRGCs and rods contribute to the color appearance in foveal vision^32^. Overall, it is necessary to match the five stimuli; L-, M-, and S-cones, rods, and ipRGCs.

Previous studies have not been able to solve the underlying problem of mismatch between cross-media because they have not rigorously examined the physical effects. In other words, the physical and psychological factors cannot be made independent, and as a fundamental problem, it cannot be denied that the difference in physical luminescence also affects the color appearance. Therefore, we need to determine whether we can match the color appearance when we compare isomeric color-matching conditions that have the same spectral distributions between cross-media. Such an isomeric condition has one more advantage in that we do not have to consider the individual differences between observers. However, it is extremely difficult to create isomeric color pairs using common luminaires because the colors are on different media with different features, depending on whether they are self-luminous or surface colors. Some researchers have developed multi-primary displays to increase the accuracy when recreating colors. Yamaguchi et al.^33^ used six primary colors and Murakami et al.^34^ used seven primary colors to reproduce a color, and created a light condition that was approximated to the spectra of an illuminated printed color. They showed that such an approximation gives imperfect color matching because it is not a perfect reproduction of the spectral distributions. Therefore, we create an isomeric color-matching condition using special equipment to confirm whether there are physical effects between self-luminous and surface colors. Generally, many experiments have been conducted on the color appearance of different devices by fixing surface colors and changing the self-luminous colors. By contrast, we fix the self-luminous colors and change the surface colors using a multispectral light source that illuminates the paper. We also prepare pentamic-metamers that meet the super-metameric condition with five photoreceptors, including the rods and ipRGCs responses. Such pentamic-metamers enable us to determine the effects of rods and ipRGCs on the color appearance of standard observers.

## Results

### Color appearance under pentamic-metamer pair stimuli

**Figure 1:**
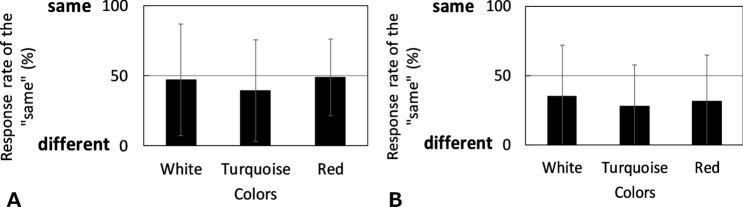
Average rates of the “same” response of thirteen people under metameric conditions. (**A**) Self-luminous color mode condition. (**B**) Surface color mode condition. The error bar indicates the standard deviation.

We examined the effect of differences in display and surface colors on the appearance of an LC display and a color patch that was illuminated by a multispectral light source. Figure 1 shows an average of thirteen participants’ response rates under the pentamic-metamer conditions. From the figure, we can conclude that an appearance match with metameric pairs cannot be achieved in cross-media color matching. Thirteen individual results are shown in Table 1 and Figure S1. Each column in Table 1 represents each stimulus, and each row represents the individual results. There were substantial individual differences among the participants, and hardly anyone could match the color appearances under pentamic-metamer conditions. It was confirmed that the color appearance between display and surface colors was mismatched in common with the previous studies^1–13^, even though the tristimulus values and rods and ipRGCs excitations were considered in this study.

**Table 1:**
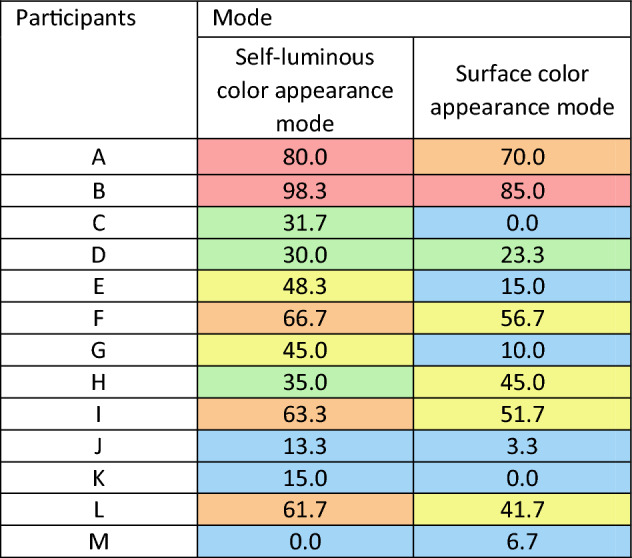
Average rates of the “same” response of the three colors under pentamic-metamer conditions in the self-luminous and surface color mode. Colors represent the response rate, Red: 100–80%, Orange: 60–79.9%, Yellow: 40–59.9%, Green: 20–39.9%, and Blue: 0–19.9%.

### Color appearance under isomeric pair stimuli

**Figure 2:**
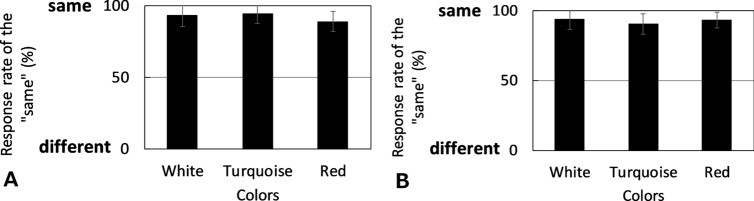
Average rates of the “same” responses of thirteen people under isomeric conditions. (**A**) Self-luminous color mode condition. (**B**) Surface color mode condition. The error bar indicates the standard deviation.

Figure 2 shows the averages of the thirteen participants’ response rates. In contrast to the results of the pentamic-metamer conditions, the isomeric conditions under both color mode conditions were over ~ 90%, which implies that we can obtain a match when the spectra between cross-media are identical. There were significant differences between isomeric and pentamic-metamer conditions (white patch: *p* < 0.001, turquoise patch: *p* < 0.001, red patch: *p* < 0.001, chi-square test). Table 2 and Figure S2 show the thirteen individual results under isomeric conditions; from the table and figure, we can conclude that all participants can match color appearance under isomeric conditions. These results show that the difference in the physical mechanism does not affect the color appearance problem. They further suggest that differences in spectral distributions cause mismatch of color appearance in cross-media color reproduction.

**Table 2:**
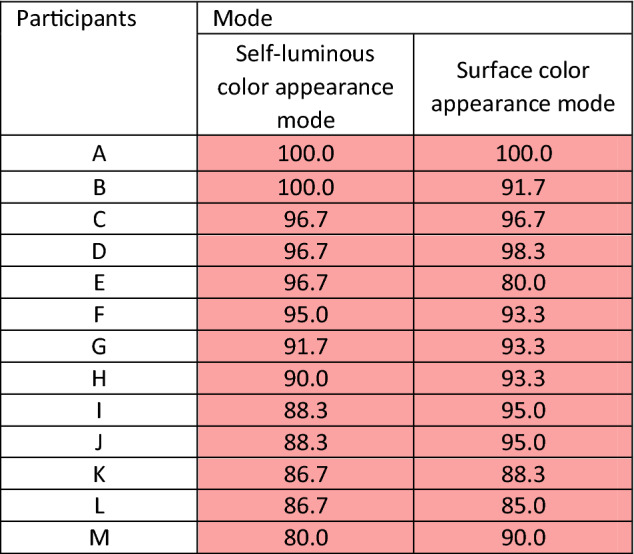
Average rates of the “same” response of the three colors under isomeric conditions in the self-luminous and surface color mode.

### Individual differences

Compared to the results under pentamic-metamer and isomeric conditions for the same participants, some participants’ scores were lower than 10% under pentamic-metamer conditions, and over ~ 90% under isomeric conditions. Participant B’s score was high under all conditions, suggesting that he/she was a standard observer by chance, whereas the others were not.

### Difference in color appearance mode

When comparing the color matching rate under pentamic-metamer conditions, the surface color mode condition was slightly lower than that of the self-luminous color mode condition (white patch: *p* < 0.01, turquoise patch: *p* < 0.01, red patch: *p* < 0.001, chi-square test). This means that we can more easily discriminate the color appearance in the surface color appearance mode than in the self-luminous color appearance mode. However, this tendency was only applied to the metameric conditions. In other words, if the spectral distributions are matched, color matching is possible in any color appearance mode.

## Discussion

To solve the problem of color appearance mismatch between cross-media, we conducted an experiment focusing on the fact that the physical properties of the devices were different. As a result, the color appearances under pentamic-metamer conditions were not matched, even though the tristimulus and excitation of rods and ipRGCs were the same. There are several possible reasons for this. First, we used the *X*, *Y*, and *Z* values of CIE1931, which were calculated from the experimental data of Guild^35^ and Wright^36^ and V(λ) of CIE1924. Judd^37^ and Vos^38^ reported a modified sensitivity function because the data were slightly lower at short wavelengths. However, even if we calculated the L, M, and S-cones using the modified data, it would be possible for the color appearances to be different because there were individual differences in CMFs^15^. This indicates that the color appearance problem cannot be explained by individual differences in cones. The second possibility is the influence of rods and ipRGCs in color appearance. It has been shown that ipRGCs have a visual impact on peripheral vision^39–43^. However, the subtypes of ipRGCs, M2 and M4, which innervate dLGN^44^, also exist in the fovea^45^, suggesting that ipRGCs affect color perception even in the fovea. Rods affect not only brightness perception but also color perception^27–29^. Therefore, the effect of rods and ipRGCs may be one of the causes. Moreover, there may be individual differences in the CMFs of rods and ipRGCs as well as cones.

In this study, we focused on cross-media color-matching problems. However, it has also been shown that reflective media versus reflective media^46^ and self-luminous versus self-luminous color appearance with different light sources^47^, displays^48^, and projectors^49^ cannot match. Such intra-mechanisms are nothing more than the difference in spectral distributions. However, this problem can be reduced to the same problem of cross-media color appearance mismatch. Our result that isomeric color matching achieves perfect appearance matching is not limited to inter-mechanism color matching (surface vs. self-luminous color); it also holds for intra-mechanism color matching (for instance, self-luminous vs. self-luminous color). However, other causes such as individual differences in CMFs or the effect of photoreceptors other than cones for intra-mechanism color matching should be considered as well as the inter-mechanism color matching.

When comparing the color appearance mode, the “same” rate of the surface color mode condition under the pentamic-metamer condition was lower than the self-luminous color mode condition (Fig. 1). It is assumed that it is more difficult to perceive the color appearance in the self-luminous color appearance mode than in the surface color appearance mode. This result corresponds to that of a previous study in which the discrimination ellipsoid of the self-luminous color appearance mode was wider than that of the surface color appearance mode^50^.

Conversely, when we observed isomeric conditions, the color appearances could be matched in both color mode conditions, even with display and surface colors (Fig. 2). It is only with this result that other studies, such as focusing on the effect of individual differences and the surrounding environment, can find significance. However, even under isomeric conditions, the “same” response rate was not 100%. We assume that the difference from 100% arises because it occurred stochastically. In a previous report^51^, the different rate of color appearance when comparing the same wavelength with stimulus onset asynchrony was equal to 0 ms, and there were approximately 40% difference evaluations. This means that when we compare the same stimuli, accidental errors arise stochastically. There might also be some fluctuations on the display and the multispectral light source; although we carefully calibrated these. Therefore, in our study, the lowest score of 80% within the error was considered. We also focused on the color appearance mode, and there was no difference under isomeric conditions. Therefore, isomeric color matching holds true universally.

It is difficult to perfectly match the spectral power distribution between different devices. Previous studies^33,34^ have attempted to reproduce some spectral distributions with multi-primary displays that used more than three primary colors. They measured the spectral reflectance of an object and displayed similar spectral distributions for the object with seven primary colors on a monitor. However, the average color difference in these studies was 0.99–1.49. Generally, we can discriminate colors when the color difference (ΔE*) is greater than 1.2 if the targets are placed side-by-side, and over 2.5 if the targets are arranged separately^52^. However, we succeeded in isolating the color appearance cross-media using a multispectral light source system and demonstrated that there is no physical cause behind the difference in color appearance.

In this study, it was experimentally proven that there was no essential problem in cross-media color matching, and isomeric color matching could perform perfect appearance even between display and surface colors. Our results are significant not only for color science, but also for industry, and lead to the importance of multispectral displays for recreating color images precisely. However, it should be noted that our results are true only in the case when no stray light falls on the target objects because the light affects the appearance.

## Methods

### Participants

Thirteen Yokohama National University students participated in the study (24.6 years ± 4.4, male: 9, female: 4). All thirteen participants repeated the experiment twenty times for all experimental conditions. We used G*Power software to estimate the sample size, and the results revealed that a sample of eleven participants could detect the effect size with *ϕ* ≥ 0.30 (statistical power = 0.95). One of the participants was an author, AK (29 years, female), of this paper. Color vision was confirmed to be normal in all participants using the Ishihara color plate, a Farnsworth–Munsell 100 Hue test, and an anomaloscope (OT-11, NEITZ, Tokyo, Japan). According to the Yokohama National University Committee on Life Science Research guidelines, this study protocol was exempted from a formal ethics review. All participants consented to the experiments in accordance with the Yokohama National University Rules on Life Science Research and provided written certificates of consent.

### Visual stimuli

**Figure 3:**
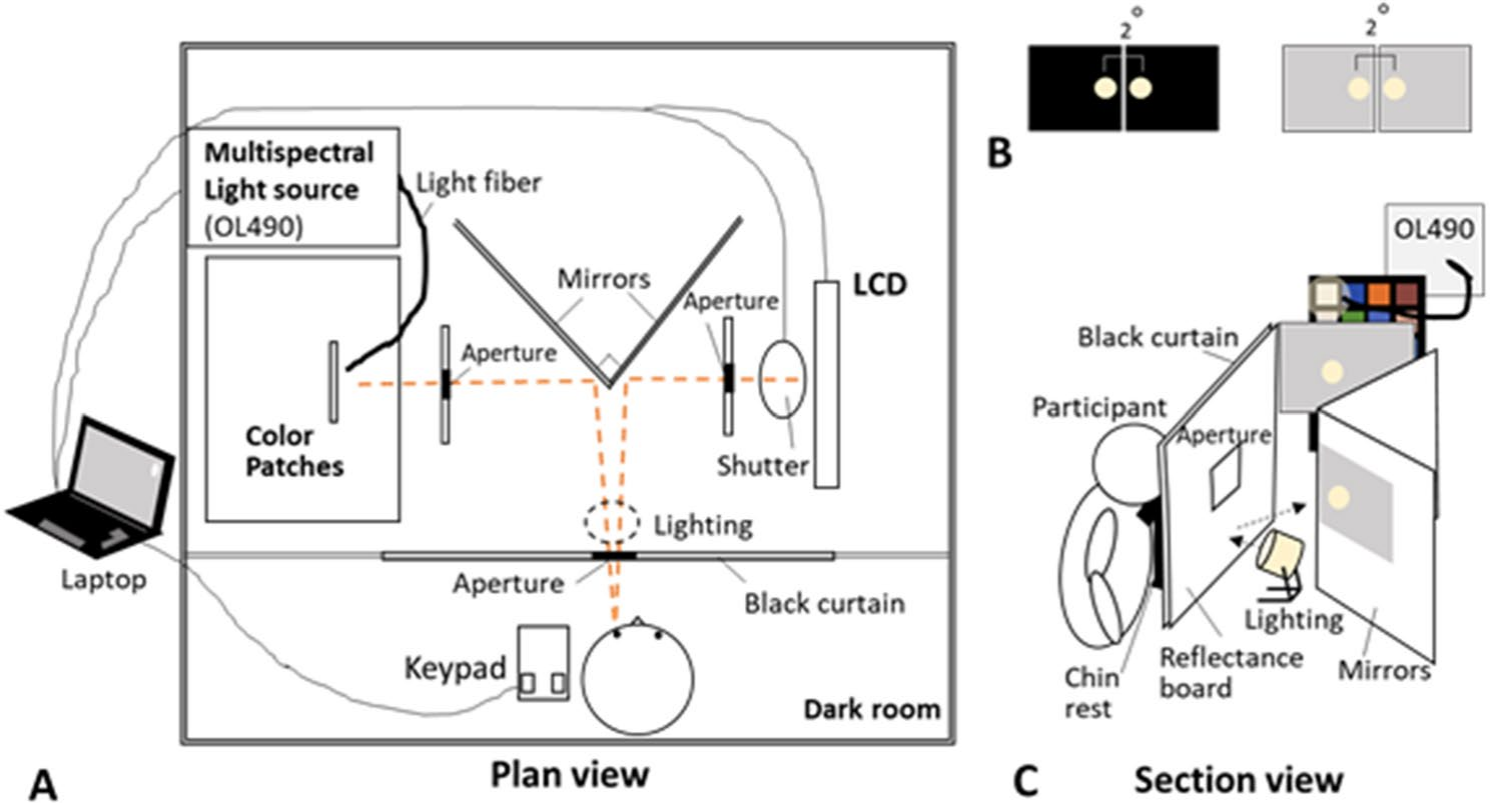
Experimental setup. (**A**) Plan of the experimental space. Mirrors were set at right angles to each other. The self-luminous color (LC display) and surface color (lighten by OL490) were alternately displayed using a shutter set in front of the display and function of OL490. The light originating from OL490, the LC display, and the shutter was controlled by a laptop that was set outside the experimental room. Participants were placed in a dark room, and observed stimuli reflected by mirrors with their left eye. The lighting was set between the reflectance board and the mirrors for the surface color appearance mode. Two boards were set between the mirrors and the color checker, and the mirrors and the shutter to display the background color in the surface color appearance mode. (**B**) Stimuli from the participants’ views. The left image shows the self-luminous color appearance mode, whereas the right image shows the surface color appearance mode. The left circle of each mode is the surface color (color checker), whereas the right circle is the self-luminous color (LC display). Both sizes are 2°, and the distance between the two is 1°. They are presented alternately. (**C**) Section of the experimental space. Lighting was set for the surface color mode condition. The light switched toward the reflectance board lighted two gray boards, and was only used for the surface color mode condition; it was turned off during the self-luminous color mode condition.

The spectral distributions of the visual stimuli were measured using a spectral meter (SR-LEDW-5N; Topcon Corporation, Tokyo, Japan). Visual stimuli for the self-luminous color condition were set on an LC display (RDT233WLM; Mitsubishi, Tokyo, Japan), whereas those for the surface color condition were set on a white color patch that was illuminated by a Digital Light Processing (DLP) Digital Mirror Device (DMD)-based multispectral light source (OL490 Agile Light Source; Gooth & Housego, Florida, USA) (Fig. 3). There was a 350-µm slit between a xenon lamp and the DMDs. All stimuli were broadband but composed of multiple spectra with an 8 nm half-bandwidth wavelength. The half-bandwidth wavelength and output intensity of each wavelength were controlled by an application written in C+ + CLI. There were two shutters in the experimental apparatus. One was to block the light from the LC display; the other was mounted inside the OL490. The two light stimuli were alternately blocked by controlling the shutters using software. Before starting the experiments, we carried out an initial aging at least for an hour. Moreover, we continued fine-tuning the stimuli and confirmed that the light source could output the spectral distributions of the target. Therefore, the stimuli in our experiments were sufficiently stable during the experiment.

**Figure 4:**
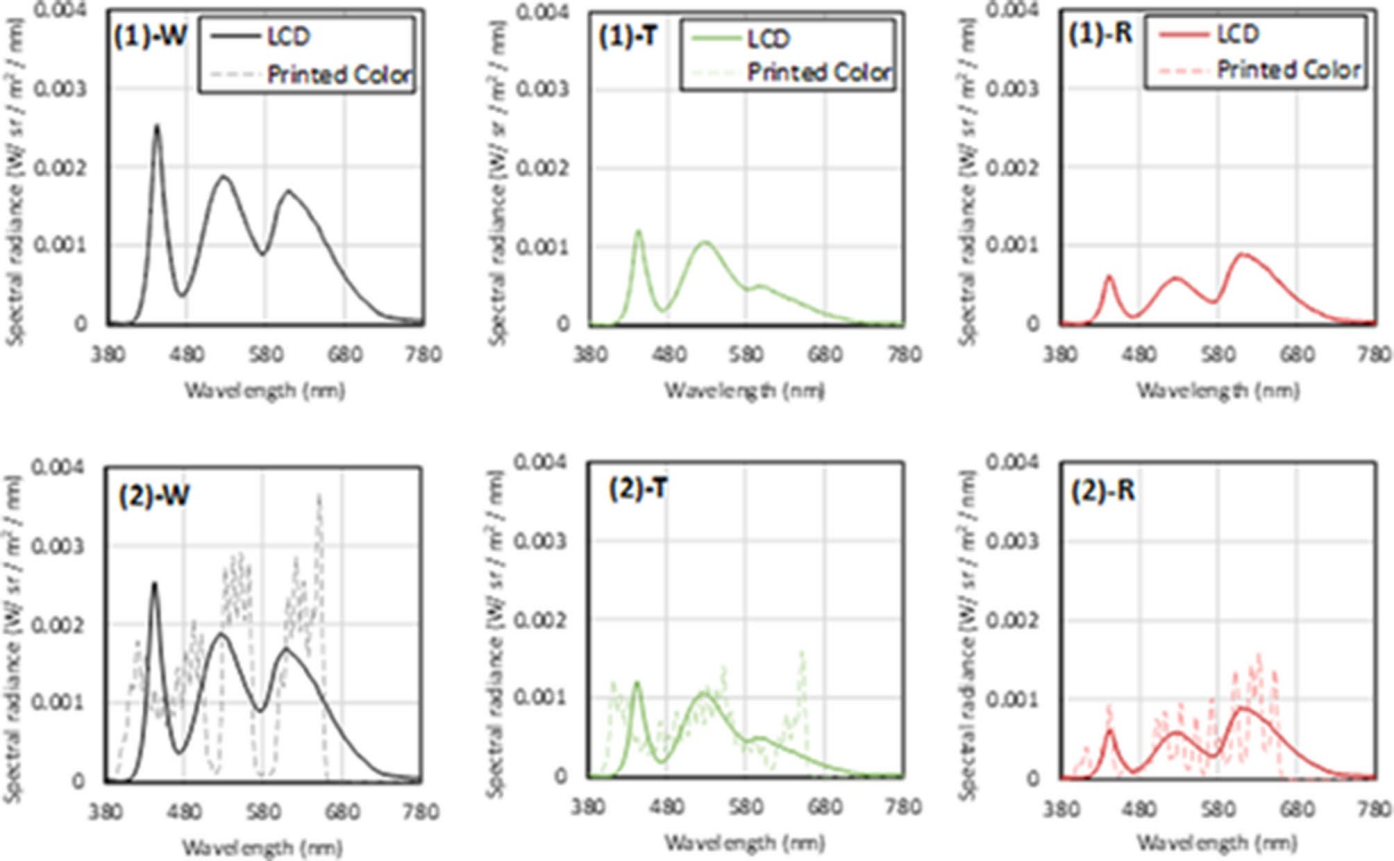
Spectral distributions of visual stimuli. (1) Isomeric, (2): Pentamic-metamer conditions. “W”: White patch, “T”: Turquoise patch, “R”: Red patch. Solid line: spectral distribution of an LC display, dotted line: spectral distribution of a surface color. These spectra were the same in the self-luminous and surface color mode conditions, and were composed of 8-nm half-bandwidth wavelengths.

Both stimuli, surface and self-luminous colors, were circular with a viewing angle of 2°. The stimuli had two conditions: (1) isomeric color-matching condition, where the spectral distributions of the display and the color patch were identical, and (2) the pentamic-metamer color matching condition, where the tristimulus values and rods and ipRGCs excitations of the two devices were identical (Fig. 4). The tristimulus values were calculated based on CIE 1931 because these values are commonly used in the industry. The spectral sensitivities of rods and ipRGCs were calculated using a pigment template^53^. The peak wavelengths of the rods and melanopsin were reported by Dartnall^54^ and Dacey^23^ to be 496.3 nm and 482 nm, respectively. Their photopigment optical densities were 0.4^55^ and 0.1^56^, respectively. The lens age was set to 32 years^14^; the macular pigment density^57^ was considered because our experiments were conducted in foveal vision (2°).

To create visual stimuli with the same tristimulus values between surface and self-luminous colors, color stimuli were chosen from 24 color patches (6 achromatic and 18 chromatic colors) in a color checker (ColorChecker Classic; X-rite, Tokyo, Japan), which is often used for color management; a lighting condition (5000 K, 500 lx, LEEM-20083N-01; Toshiba; Tokyo, Japan) was also set. Only 12 out of 18 chromatic colors under the lighting condition can be reproduced in the LC display because the color gamut that can be displayed on the LC display was limited. Three color stimuli were chosen out of these 12 chromatic colors and 6 achromatic colors (Table 3). In the case of white patch, there were the most differences of the ipRGCs and rods responses between a display and a surface color. The turquoise patch had the highest response of ipRGCs and rods in the 12 patches, whereas the red patch had the lowest response. The surface colors were generated directly by shining light from the OL490 on the white color patch.

**Table 3:** Actual measured values for each condition.

Conditions	*X*	*Y*	*Z*	*rods*	*ipRGCs*	*x*	*y*
**White patch**							
LC display	96.5	100.3	81.9	74.8	70.9	0.346	0.359
Isomeric	96.2	99.6	81.4	74.1	70.2	0.347	0.359
Metameric	97.3	100.5	82.3	74.9	71.6	0.347	0.358
**Turquoise patch**							
LC display	36.6	47.7	39.8	40.8	38.4	0.294	0.384
Isomeric	37.0	48.0	40.2	41.0	38.7	0.295	0.383
Metameric	36.6	48.0	39.9	41.0	38.5	0.293	0.385
**Red patch**							
LC display	40.0	36.5	20.3	22.7	20.9	0.413	0.377
Isomeric	40.0	36.6	20.3	22.8	20.9	0.412	0.377
Metameric	40.4	37.0	20.3	22.2	21.3	0.413	0.378

To confirm whether the isomeric color matching could perform not only self-luminous color appearance mode but also surface color appearance mode, two background colors, black and gray, were also set for the experimental condition so that the color appearances were under a self-luminous color appearance mode and a surface color appearance mode, respectively^58^. A light source (LDR 14N-W; Toshiba; Tokyo, Japan) was placed between the reflectance board and mirrors, and the color appearance mode was switched on/off by using light. In the surface color appearance mode, the light source was turned toward the reflectance board, which uniformly lightened the background of the target. Therefore, the spectrum distributions of the surface color mode condition were the sum of the spectrum of the light for the background and that of the color on display or surface color. The spectrum distributions of the surface color mode condition were set such that the spectrum distributions were the same as those of the self-luminous color mode condition by adjusting the output power of OL490. The luminosity of the background in the surface color mode condition was 80 cd/m^2^, which was sufficient to enable viewing of the surface color appearance mode^59^, and that in the self-luminous color mode condition was below 0.1 cd/m^2^. Moreover, by using a 2D spectroradiometer (SR-5000 HWS/ TOPCON TECHNOHOUSE), it was confirmed whether the background 2D distributions of the targets were different. Finally, the lighting did not directly enter the participant’s eyes.

### Procedure

**Figure 5:**
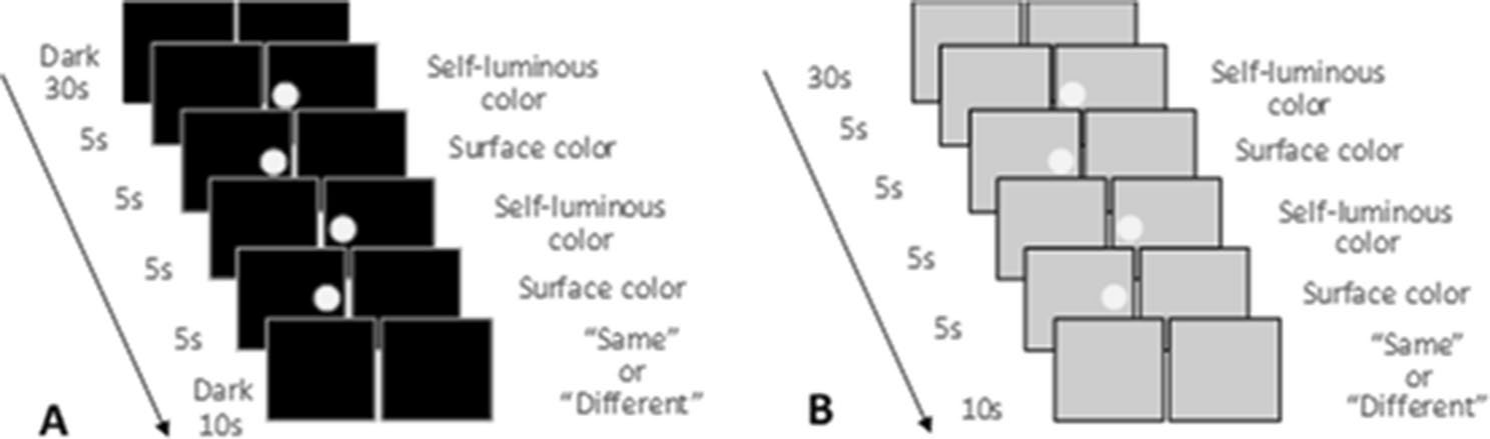
Presentation sequence of surface color and self-luminous color. (**A**) Self-luminous color mode condition. (**B**) Surface color mode condition.

The experiment was conducted to determine whether the color appearances were the same between the display color and the color patch. The flow of the experiments between each color mode condition was the same (Fig. 5). First, in a dark room, the participants observed, through an aperture, the space where the targets were displayed. The space was dark (for the self-luminous color appearance mode) or illuminated only the background (for the surface color appearance mode) for 30 s before the experiment as resting time. After a beep sound, each color on the two devices, display or color patch, was displayed alternately for 5 s and repeated twice. This is to prevent any simultaneous contrast effect and to control the gaze duration. Participants observed these stimuli with their left eye at foveal vision with an eye mask to cover their right eye. Finally, participants evaluated whether the appearance of the two light stimuli were the “same” or “different,” using a keypad. There were 10 s between each trial, and the trials were repeated 20 times per condition. Isomeric and pentamic-metamer conditions were displayed in random order, and an experiment of each color appearance mode was conducted separately. None of the participants were informed that the two circles were displayed in different ways.

## Supplementary information

Supplementary Information

## Data Availability

The datasets generated and analyzed during the current study are available from the corresponding author upon reasonable request.
