# Supplementary material for: Perfect appearance match between self-luminous and surface colors can be performed with isomeric spectra
Source: Sci Rep. 2020 Oct 27;10:18350. doi: 10.1038/s41598-020-75510-x (PMC7591860; doi:10.1038/s41598-020-75510-x)
Supplement: Supplementary file 1 — Supplementary Information [file 41598_2020_75510_MOESM1_ESM.docx]

**Perfect Appearance Match between Self-luminous and Surface Colors Can be Performed with Isomeric Spectra**

Akari Kagimoto^1,*^ and Katsunori Okajima^2^

^1^Graduate School of Environment and Information Sciences, Yokohama National University, Yokohama 240-8501, Japan
^2^Faculty of Environment and Information Sciences, Yokohama National University, Yokohama 240-8501, Japan

Supplementary Information

**A**

**B**

Figure S1. Individual results under the pentamic-metamer conditions. (A) Self-luminous color mode condition. (B) Surface color mode condition.

**A**

**B**

Figure S2. Individual results under the isomeric conditions. (A) Self-luminous color mode condition. (B) Surface color mode condition.
